# Acute Alcohol Drinking Promotes Piecemeal Percepts during Binocular Rivalry

**DOI:** 10.3389/fpsyg.2016.00489

**Published:** 2016-04-06

**Authors:** Dingcai Cao, Xiaohua Zhuang, Para Kang, Sang W. Hong, Andrea C. King

**Affiliations:** ^1^Department of Ophthalmology and Visual Sciences, University of Illinois at Chicago, ChicagoIL, USA; ^2^Department of Psychology and Center for Complex Systems and Brain Sciences, Florida Atlantic University, Boca RatonFL, USA; ^3^Department of Psychiatry and Behavioral Neuroscience, The University of Chicago, ChicagoIL, USA

**Keywords:** binocular rivalry, piecemeal percept, acute alcohol effect

## Abstract

Binocular rivalry refers to perceptual alternation when two eyes view different images. One of the potential percepts during binocular rivalry is a spatial mosaic of left- and right-eye images, known as piecemeal percepts, which may result from localized rivalries between small regions in the left- and right-eye images. It is known that alcohol increases inhibitory neurotransmission, which may reduce the number of alternations during binocular rivalry. However, it is unclear whether alcohol affects rivalry dynamics in the same manner for both coherent percepts (i.e., percepts of complete left or right images) and piecemeal percepts. To address this question, the present study measured the dynamics of binocular rivalry before and after 15 moderate-to-heavy social drinkers consumed an intoxicating dose of alcohol versus a placebo beverage. Both simple rivalrous stimuli consisting of gratings with different orientations, and complex stimuli consisting of a face or a house were tested to examine alcohol effects on rivalry as a function of stimulus complexity. Results showed that for both simple and complex stimuli, alcohol affects coherent and piecemeal percepts differently. More specifically, alcohol reduced the number of coherent percepts but not the mean dominance duration of coherent percepts. In contrast, for piecemeal percepts, alcohol increased the mean dominance duration but not the number of piecemeal percepts. These results suggested that alcohol drinking may selectively affect the dynamics of transitional period of binocular rivalry by increasing the duration of piecemeal percepts, leading to a reduction in the number of coherent percepts. The differential effect of alcohol on the dynamics of coherent and piecemeal percepts cannot be accounted for by alcohol’s effect on a common inhibitory mechanism. Other mechanisms, such as increasing neural noise, are needed to explain alcohol’s effect on the dynamics of binocular rivalry.

## Introduction

Binocular rivalry refers to perceptual alternations between two different images presented simultaneously to the two eyes (Blake and Logothetis, 2002). Neural mechanisms mediating binocular rivalry have been the center of debate for many decades. The general consensus is that binocular rivalry is mediated by neural competition that occurs at multiple stages in the visual hierarchy (Blake and Logothetis, 2002; Wilson, 2003; Tong et al., 2006; Alais, 2012). One of the neural models for binocular rivalry posits that reciprocal inhibition between visual neurons representing left- and right-eye images and self-adaptation in neural signals determine the dynamics of rivalry (Lehky, 1988; Blake, 1989; Wilson, 2003, 2007). Consistent with this model, it has been demonstrated that a higher brain concentration of the inhibitory neurotransmitter gamma-aminobutyric acid (GABA) was associated with a low alternation rate in bi-stable percepts, including binocular rivalry (van Loon et al., 2013). In addition, it has been shown that visual adaptation (Blake et al., 2003; Alais et al., 2010; Kang and Blake, 2010; Theodoni et al., 2011) and attention (Mitchell et al., 2004; Chong et al., 2005; Chong and Blake, 2006; Paffen et al., 2006; Hancock and Andrews, 2007; Zhang et al., 2011) are critical determinants of binocular rivalry dynamics.

When two dissimilar images presented to the two eyes are relatively large, in addition to the coherent percepts of the left- or right-eye images, one can also experience piecemeal percepts, in which patches of left- and right-eye images are visible simultaneously (Kovács et al., 1996; Polonsky et al., 2000; Lee and Blake, 2004). Previous studies show that piecemeal percept rarely occurs if the dissimilar images are smaller than about 7 min of arc in visual angle at fovea (Blake et al., 1992), indicating that binocular rivalry occurs at local regions and the perceptual outcome during rivalry depends on local competitions. In other words, the coherent perception of a left- or right-eye image likely requires joint predominance of local rivalries (Fries et al., 1997; Alais and Blake, 1999).

Are piecemeal percepts during binocular rivalry also mediated by the same inhibitory/adaptation mechanism as coherent percepts? Despite the extensive efforts devoted to understand the mechanisms mediating perceptual switches between coherent rivalrous images during binocular rivalry, the mechanism for piecemeal percepts is less clear. It has been shown that the principles of Gestalt perceptual grouping, such as feature similarity and good continuation, can affect the joint predominance of local rivalries (Kovács et al., 1996; Alais and Blake, 1999; Stuit et al., 2011). Besides this grouping-based account, computational models have been developed to account for piecemeal percepts. For instance, Stollenwerk and Bode (2003) assumes that multiple neurons represent different spatial zones in the images and those representations of corresponding zones in the two-eye images compete with each other through inhibition and adaptation. When the dominant patterns differ among different zones, piecemeal percepts occur. In addition, piecemeal percepts are considered as a result from the transitional period between the two coherent percepts for the left- and right-eye images and neural noise has been suggested to play a critical role in resolving rivalry during the transition period between two rivalrous percepts (Brascamp et al., 2006; Kang and Blake, 2010).

Acute alcohol drinking is known to increase inhibition in the central nervous system by increasing inhibitory neurotransmission or by inhibiting excitatory neurotransmission (Valenzuela, 1997; Grobin et al., 1998). Therefore, acute alcohol administration can be thought as a pharmacological manipulation of the inhibitory system that can affect binocular rivalry dynamics. Studies have shown that acute alcohol consumption reduces the alternation rate between left- and right-eye percepts during binocular rivalry, consistent with alcohol’s increase in neural inhibitory effects (Barany and Hallden, 1947; Donnelly and Miller, 1995). These studies, however, have focused on coherent percepts only and have not considered the influence of alcohol consumption on piecemeal percepts. This is important as the knowledge of alcohol’s effect on the dynamics of piecemeal percepts may provide insights in the mechanisms for the dynamics of binocular rivalry. We hypothesized that if a common (inhibition) mechanism determines perceptual experiences of both coherent and piecemeal percepts during binocular rivalry, alcohol would affect the rivalry dynamics in the same manner for both coherent and piecemeal percepts.

## Materials and Methods

### Participants

We focused on testing young moderate and heavy social drinkers, because this population can tolerate the alcohol dose used in the study without significant adverse effects and they are at risk for alcohol-related harm but have not incurred significant withdrawal or other clinical symptoms that might confound our measurements (Caetano et al., 1998). Young moderate-to-heavy social drinkers were recruited via internet advertisements and were screened using online screening questionnaires and interview, which included demographic information, medical information (eye disease, heart disease, diabetes, high blood pressure, mental health, etc.), the Alcohol Quantity-Frequency Interview (Cahalan et al., 1969) and the Timeline Follow-back calendar (Sobell et al., 1979) for daily estimates of alcohol drinking. Inclusion criteria were: having normal or corrected-to-normal acuity, not reporting any health and psychiatric problems including alcohol dependence that might interfere with the study procedures, consuming at least 6 or more alcoholic drinks weekly (up to 35) and engaging in binge drinking [consuming 5+ drinks/occasion for men and 4+ for women (Substance Abuse and Mental Health Services Administration [SAMHSA], 2005] at least twice monthly up to four times weekly.

There were 15 participants [six males and nine females, age 25(*mean*) ± 2.4(*SD*) years; number of drinking days per month: 11.5 ± 5.2; number of standard drinks per drinking day: 1.3 ± 0.4; number of binge days per month: 4.4 ± 2.2; maximum number of drinks consumed on one occasion: 7.7 ± 2.9]. This study was approved by the University of Illinois at Chicago Institutional Review Board and was in compliance with the Declaration of Helsinki.

### Overall Design and Protocol

The experiment used a within-subject, double-blinded and placebo-controlled design. Each participant received either an intoxicating dose of alcohol (0.8 g/kg, i.e., equivalent to 4–5 standard alcohol drinks (King et al., 2011; Zhuang et al., 2012) or placebo beverage on two separate days. The alcoholic beverage consisted of 16% volume ethanol, which contained 190-proof ethanol mixed with water, grape-flavored drink mix and a sucralose-based sugar substitute. The placebo beverage included 1% volume ethanol as a taste mask to reduce expectancy effects. Participants drank the assigned beverage through a straw from a plastic, lidded cup to help conceal the scent and identification of the alcohol content. Women received 85% of the dose of men as a correction for body water differences (Watson et al., 1980). The total beverage volume was (*mean*) 435.3 ± (*SD*) 81.6 ml and divided into two equal portions.

The order of the two beverage administrations was randomized, with a minimum of a 48-hour interval between the sessions. The participants were instructed to abstain from alcohol and recreational drugs for 48 hours prior to each session, and both participant and experimenter were double-blinded to the beverage content. To verify alcohol abstinence, breath alcohol concentration (BrAC) was measured using an Alco-Sensor IV (Intoximeter Inc., St. Louis, MO, USA) upon arrival in each experimental session. Following BrAC measurement, the participant was provided with a light snack (non-caffeine, low-fat meal at 15% calories based on body weight) and then taken to a dark room for the binocular rivalry experiment. The participants received a tutorial and practice period in the first session, followed by the pre-beverage assessment. After this assessment, the participants drank the assigned beverage. They had 5 min to consume the first half portion, followed by a 5-min rest period, and then another 5 min to finish the second half portion. Post-beverage measurements of binocular rivalry were conducted 50 minutes after completing beverage intake. BrAC levels were measured before the binocular rivalry experiment during pre- and post-beverage assessments. This beverage administration procedure has been used extensively in previous studies and has shown reliable rising and declining breath alcohol concentration (BrAC) curves across participants (Brumback et al., 2007; King et al., 2011; Zhuang et al., 2015). The BrAC levels were all zero at baseline for both sessions, confirming recent alcohol abstinence in all participants. Post-beverage BrAC levels, measured right before the binocular rivalry testing, were at 0.071 ± 0.011 g/dl in the alcohol session or at zero in the placebo session.

### Apparatus

Stimuli were generated using an Apple iMac computer and presented on a calibrated NEC cathode ray tube (CRT) display (MultiSync FE 991 SB). The display had 1600 × 1200 pixel resolution and a refresh rate of 60 Hz noninterlaced. Different stimuli were presented to each eye by projection through a mirror haploscope. The positions of the mirrors were adjusted for each participant in order to compensate for the differences in interpupillary distance.

### Visual Stimuli

**FIGURE 1:**
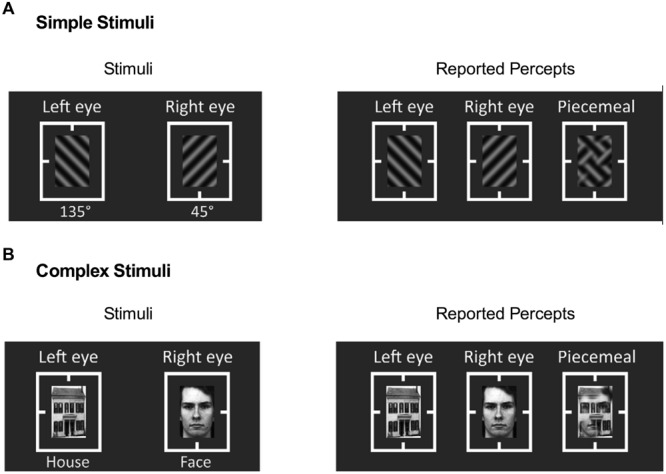
**Binocular rivalry stimuli and reported percepts in Experiments 1 and 2. (A)** Simple stimuli, gratings with two different orientations, and **(B)** Complex stimuli, house and face images. Reported percepts comprised of left-eye image, right-eye image, and piecemeal of left- and right-eye images.

As prior studies have examined alcohol’s effects on only simple geometric stimuli such as sine-wave gratings, it is unclear whether alcohol affects binocular rivalry with complex recognizable stimuli (e.g., between a house and a face). Such complex recognizable stimuli are perceived longer during binocular rivalry (Hastorf and Myro, 1959; Yu and Blake, 1992), indicating discrepancy in rivalry dynamics between simple geometric stimuli and complex recognizable stimuli. Thus we used two sets of stimuli in the main experiment (**Figure 1**), including: (1) simple stimuli that consisted of two orthogonal sinusoidal gratings (45° vs. 135°, **Figure 1A**) at the same spatial frequency of 4 cycle/degree, and (2) complex stimuli that consisted of a house and a face (**Figure 1B**). The size of all stimuli was 1°×1.5°. Several thin rectangular lines guided fixation. The mean luminance of all stimuli was 23.5 cd/m^2^. The root mean square (RMS) contrasts of all stimuli were kept constant and minimum and maximum luminance were 5 and 42 cd/m^2^, respectively, leading to a Michelson contrast of 79%.

### Binocular Rivalry Measurement Procedure

Each experiment began with the presentation of the rectangular thin, fusion guidelines. The participants adjusted the mirrors in order to get good alignment of left- and right-eye images. When ready, the participants pressed a button in a Gamepad to start a trial, during which both the left- and right-eye stimuli were presented continuously for 40 seconds. The participants reported their perceptual experience (either one of the coherent percepts or piecemeal percept) by continuously holding a button until the percept changed. Separate buttons were assigned to report three different percepts in the experiment: percept of the left-eye stimulus, percept of the right-eye stimulus, and piecemeal percept (**Figure 1**, right column). The duration of each percept was measured by taking the duration of holding a designated button. Once a trial was completed, the participants could rest and pressed a button to start the next trial. Each experiment consisted of eight trials (two stimulus types × four repeats), which were presented in a random order. For each stimulus type, the presentation was counterbalanced across the left and right eyes. For example, for the simple stimuli, the 45° grating were presented to the left eye in two trials and to the right eye in the other two trials. Each experiment lasted for approximately 15 min. Each participant completed four binocular rivalry measurements (pre-placebo, post-placebo, pre-alcohol, and post-alcohol).

### Statistical Analysis

To simplify the analysis, the reported percepts were grouped into two categories, coherent percepts (i.e., perceiving the left- or right-eye image) and piecemeal percepts. For each trial, we calculated the time to rivalry onset (Casanova et al., 2013), the first percept category and duration, the total number of percepts (coherent and piecemeal percepts combined), and percept-specific total dominance duration (i.e., total time perceiving a specific percept in a trial), the number of percepts and mean dominance duration. The mean dominance duration was computed as dividing the total dominance duration by the number percepts for each category of percepts (coherent and piecemeal). Trials with time to rivalry onset ≥5 s (account for 2.3% of the total number of trials) were removed. For each test condition (Stimulus Type × Beverage Type × Time combination), percepts with the largest 2% and lowest 2% of mean dominance durations were excluded from analyses to avoid percepts with extremely short or long duration.

**FIGURE 2:**
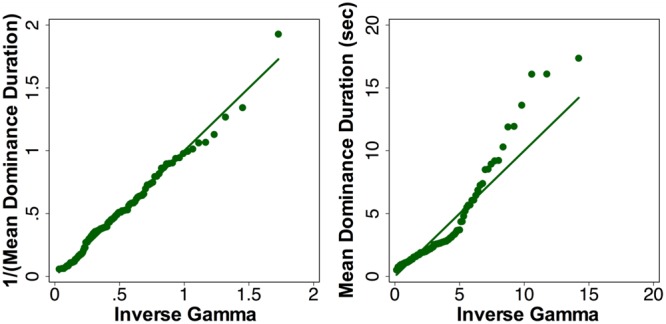
**The quantiles of the reciprocal of mean dominance duration **(left)** or mean dominance duration **(right)** versus the quantiles of a fitted gamma distribution for pre-beverage measurement during the placebo session with the simple grating stimuli.** A gamma distribution described the reciprocal of mean dominance duration better than the dominance duration.

The time to rivalry onset, first percept category and duration, the number of percepts and dominance duration were analyzed using Generalized Estimation Equation (GEE) (Zeger and Liang, 1986) models that could account for within-subject correlations among repeated measurements and have a flexibility in fitting data with various distributions. We used a Gaussian distribution for modeling the log-transformed time to rivalry onset and log-transformed total dominance duration, a Binomial distribution for the first percept category and a Poisson distribution for modeling the number of percepts. It has been suggested that the reciprocal of dominance duration instead of the mean dominance durations follows a Gamma distribution (Brascamp et al., 2005) and we confirmed this is the case (see **Figure 2** for the pre-beverage rivalry data with the simple stimuli during the placebo session). Therefore, we calculated the reciprocal transformation for mean dominance duration and used a gamma distribution in the GEE modeling. For the time to rivalry onset, first percept category, and the total number of percepts, the GEE model included Stimulus Type (simple vs. complex stimuli), Beverage Type (placebo vs. alcohol), Measurement Time (pre- vs. post-beverage), and their interactions. For percept-specific measures (first percept duration, total dominance duration, number of percepts and mean dominance duration), the GEE models included Stimulus Type (simple vs. complex stimuli), Percept Category (coherent vs. piecemeal), Beverage Type (placebo vs. alcohol), Measurement Time (pre- vs. post-beverage), and their interactions. Following each of the GEE models, we used linear contrast tests to test the model terms (main effects or interactions). We were primarily interested in the significance of several interaction terms in the models, including (1) Beverage Type × Time to test the alcohol effect, (2) Percept Category × Beverage Type x Time to test whether the alcohol effect depended on the perception category, and (3) Stimulus Type x Beverage x Time or Stimulus Type × Percept Category × Beverage × Time to test whether the alcohol effect depended on the stimulus types. In the case that one of the above mentioned interaction terms was significant, we then conducted post-estimation pairwise comparisons to compare pre-beverage and post-beverage values within a session for each stimulus type at the Bonferroni corrected significance level of 0.0125 (i.e., four post-estimation comparisons for each stimulus type).

## Results

**FIGURE 3:**
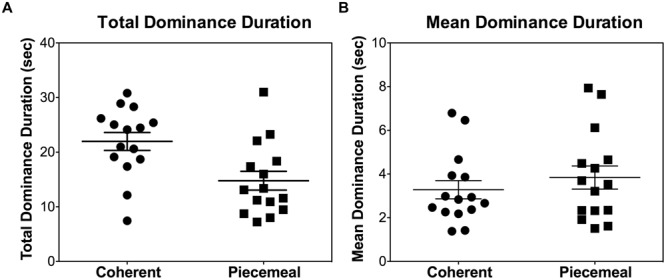
**Individual differences in **(A)** total dominance duration per trial (40 s) and **(B)** mean dominance duration for each of the participants during the placebo session, collapsing the stimulus types and measurement times.** Each symbol represents one individual.

There were large individual differences in terms of total dominance duration or mean dominance duration for coherent and piecemeal percepts (see **Figure 3** rivalry data during the placebo session, collapsing stimulus types and measurement time), with some subjects predominantly perceiving coherent percepts while others had a more balanced coherent and piecemeal predominance. Note that the participants in our study were moderate-to-heavy alcohol drinkers (in contrast, typical binocular rivalry studies used normal subjects probably with a light drinking pattern). It is known that chronic alcohol exposure may change the balance of inhibitory and excitatory processes in the brain (Valenzuela, 1997), potentially leading to a large individual difference. However, our sample size was not large enough to assess the association between drinking history and predominance pattern so this issue was outside of the scope of this paper.

**FIGURE 4:**
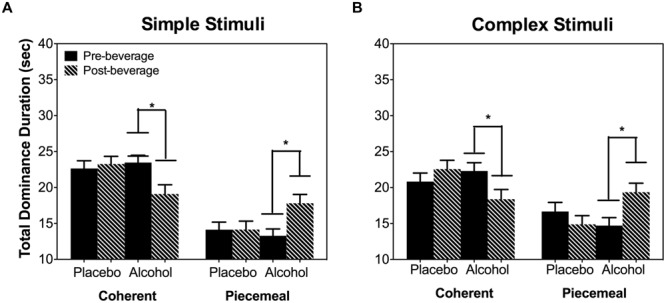
**Total dominance duration per trial (40 s) of coherent or piecemeal percepts for the simple stimuli **(A)** and complex stimuli **(B)**.** Error bars are ±SEM. ^∗^*p* < 0.0125 for paired comparisons between pre- and post-beverage measurements in the same session.

Alcohol significantly reduced the total dominance duration for coherent percepts and increased the total dominance duration for piecemeal percepts [Percept Category × Beverage Type x Time: χ^2^(1) = 21.95, *p* < 0.001; **Figure 4**] and this alcohol effect was not related to stimulus types [Stimulus Type × Percept Category × Beverage Type × Time: χ^2^(1) = 0.14, *p* = 0.707]. Subsequent pairwise comparisons for both stimulus types showed that alcohol increased post-beverage total piecemeal duration (or a decreased post-beverage total coherent duration) compared with the pre-beverage measurement but this was not the case in the placebo session (**Figure 4**).

**FIGURE 5:**
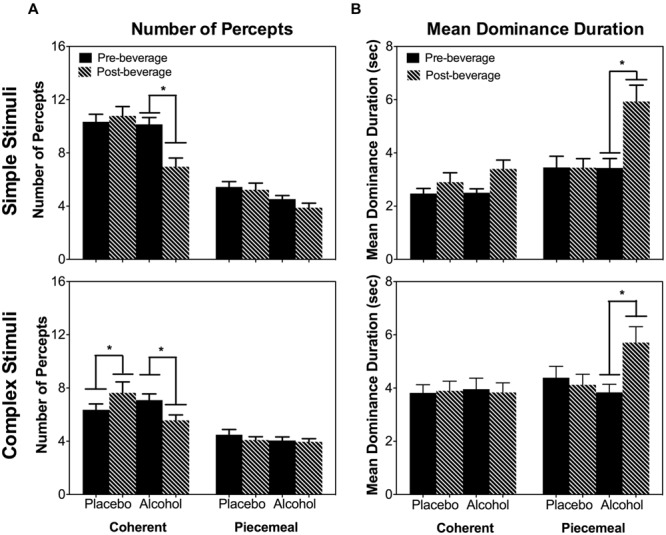
**The number of percepts **(A)** and mean dominance duration **(B)** measured at Pre- and Post-beverage for Placebo and Alcohol sessions for the two stimulus types, simple stimuli (top), and complex stimuli (bottom).** Error bars are ±SEM. ^∗^*p* < 0.0125 for paired comparisons between pre- and post-beverage measurements in the same session.

**FIGURE 6:**
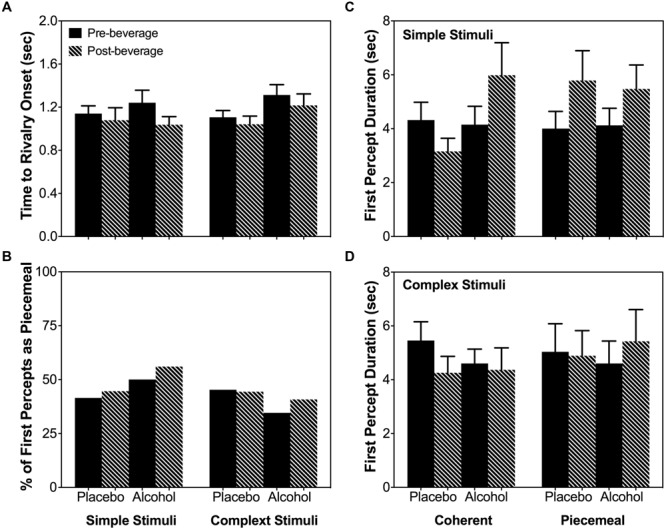
**Time to rivalry onset **(A)**, first percept category **(B)** and first percept duration for the simple stimuli **(C)** and complex stimuli **(D)**.** Error bars are ± SEM.

As the total dominance duration is determined by the number of percepts and the mean dominance duration of each percept category, we analyzed alcohol’s effect on the number of percepts and mean dominance duration separately. For both stimulus types (simple and complex), alcohol decreased the number of percepts for coherent percepts but not for piecemeal percepts [Percept Category × Beverage Type × Time: χ^2^(1) = 16.20, *p* < 0.001; Stimulus Type × Percept Category × Beverage Type × Time : χ^2^(1) = 0.93, *p* = 0.334; **Figure 5A**]. On the other hand, for both simple and complex stimuli, alcohol significantly increased the mean dominance duration for piecemeal percepts but not for coherent percepts [Percept Category x Beverage Type x Time: χ^2^(1) = 6.63, *p* = 0.01; Stimulus Type x Percept Category x Beverage Type x Time : χ^2^(1) = 0.18, *p* = 0.671; **Figure 5B**]. Finally, alcohol did not affect time to rivalry onset (**Figure 6A**), the first percept category (**Figure 6B**), or first percept duration (**Figures 6C,D**) significantly for both stimulus types. These results indicate that alcohol affects coherent and piecemeal percepts differently. That is, alcohol reduced the number of coherent percepts (without changing mean dominance duration) but increased mean dominance duration for piecemeal percepts (without changing the number of piecemeal percepts), leading to a reduction in total coherent percept duration but an increase in total piecemeal duration (**Figure 4**).

**FIGURE 7:**
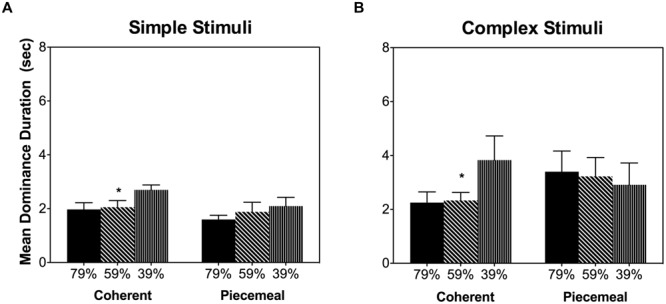
**Mean dominance duration for different percept categories in the control experiment for the simple stimuli **(A)** and complex stimuli **(B)**.** Error bars are ±SEM. ^∗^*p* < 0.0125 for post-estimation paired comparisons.

It is known that acute alcohol intake impairs contrast sensitivity (Pearson and Timney, 1998; Zhuang et al., 2012). Therefore, the alcohol’s effects on the dynamic of binocular rivalry may be in fact due to the impaired contrast sensitivity and decreased stimulus visibility from alcohol. To test this possibility, we measured binocular rivalry dynamics using the same stimuli while varying the contrasts (79, 59, or 39%, mean luminance at 23.5 cd/m^2^) in 4 lab personnel (one male and three females, age 26.8 ± 7.2 years, light drinkers) without alcohol intake. This control experiment showed that for both stimuli types, reducing contrast increased the mean dominance duration for coherent percepts but not for piecemeal percepts (**Figure 7**), indicating that a decrease in stimulus visibility could not explain the alcohol effects on piecemeal percepts (**Figure 5B**).

## Discussion

Acute alcohol intake impairs various aspects of visual processing (Khan and Timney, 2007; Kunchulia et al., 2012; Zhuang et al., 2012, 2015). Here, we investigated whether acute alcohol intake altered the dynamics of binocular rivalry, including coherent and piecemeal percepts. We hypothesized that if a common mechanism determines perceptual experiences of both coherent and piecemeal percepts during binocular rivalry, alcohol would affect the rivalry dynamics in the same manner for both coherent and piecemeal percepts. Consistent with previous studies (Barany and Hallden, 1947; Donnelly and Miller, 1995), the current study also showed that acute alcohol intake slowed down the number of percepts between seeing coherent percepts during binocular rivalry (**Figure 5A**). However, alcohol affected the dynamics of piecemeal percepts differently from coherent percepts. For coherent percepts, alcohol reduced the number of percepts but not the mean dominance duration, and for piecemeal percepts, it was the opposite (alcohol did not reduce the number of percepts but did increase the mean dominance duration). Therefore, our hypothesis was not supported.

Based on Levelt’s Fourth proposition, increasing stimulus strength (e.g., stimulus contrast) in both eyes while keeping stimulus strength equal between eyes will generally increase the alternation rate or reduce dominance duration (Levelt, 1965; Brascamp et al., 2015). Acute alcohol intake is known to impair contrast sensitivity, leading to a weaker stimulus strength (visibility) (Pearson and Timney, 1998; Zhuang et al., 2012). Therefore, alcohol could affect the dynamic of binocular rivalry through it’s reduction in contrast sensitivity. Our control experiment, however, showed that changing luminance contrast affects mean dominance duration of coherent percepts but not piecemeal percepts, suggesting that it is unlikely that alcohol’s increase in piecemeal percept duration was due to alcohol’s reduction in contrast sensitivity. Mueller and Blake (1989) showed the predominance time of piecemeal percepts did not vary with stimulus contrast in binocular rivalry, similar to our results from the control experiment. Interestingly, using a binocular motion rivalry paradigm (Platonov and Goossens, 2013), an increase in random dot coherence led to an increase in dominance duration of piecemeal percepts, a result similar to our observed alcohol’s effect, while changing stimulus contrast did not change piecemeal percept duration. Given the random-dot-coherence and contrast manipulations had different effects on rivalry dynamics, it is possible that the random-dot-coherence and contrast manipulations targeted on different mechanisms for binocular rivalry (see next paragraph for further discussion). Further, Brascamp et al. (2006) showed that a decreasing contrast led to a longer transition duration in which both superimposition (fusion) or piecemeal percepts could occur (Hollins, 1980). They reported that percepts during transitional period between left- and right-eye images were mainly superimposition (fusion) percepts with low contrast (near threshold) rivalry stimuli; while the transitional percepts were predominantly piecemeal with high contrast rivalry stimuli. In our control experiment as well the main experiment, we used a high contrast (39–79%), which were high enough for mainly seeing piecemeal percepts instead of fusion during the transitional period (Brascamp et al., 2006). In other words, the observed alcohol effects on piecemeal percepts could not be confounded with fusion percepts that we did not ask to report.

Binocular rivalry research has pointed to the importance of mutual inhibition, adaptation and neural noise in determining rivalry dynamics. Computational modeling indicates that mutual inhibition and adaptation determines the percept choice based on two eye images, while neural noise is critical for transitional period (Brascamp et al., 2006; Huguet et al., 2014). Acute alcohol drinking is known to increase inhibition in the central nervous system. An increased inhibition between the representations of two images presented to two eyes is expected increase the mean dominance duration of coherent percepts. However, our study showed that the mean dominance duration for coherent percepts did not change significantly by alcohol intake. Therefore, our results could not be accounted for by alcohol’s increase in inhibition. Previous studies have shown alcohol-induced slowdown in alternation rates when only coherent percepts were considered (Barany and Hallden, 1947; Donnelly and Miller, 1995). The current study showed that previous findings may result from the increased piecemeal percept duration rather than from a strengthened inhibition. Instead, the reduced number of coherent percepts might be resulting from the increased duration of piecemeal percepts during the transitional period. As seen in **Figures 4** and **5**, both the total predomaince time and the mean dominance duration were increased for piecemeal percepts after alcohol intake. Physiological investigations have shown that alcohol reduces signal-to-noise ratios or increases noise in the primary visual cortex (Chen et al., 2010). Given the importance of neural noise in transitional period of rivalry dynamics, it is likely acute alcohol drinking may affect the dynamics of piecemeal percepts by increasing neural noise (Brascamp et al., 2006). In sum, our study suggested that acute alcohol intake selectively increased the duration of piecemeal percepts, potentially by increasing neural noise, leading to a reduction in the number of coherent percepts.

## Author Contributions

DC and AK conceptualized the alcohol challenge paradigm. DC, XZ, PK, and SH designed the visual experiments. PK and XZ collected the data. DC and SH provided interpretation of the results. All contributed to manuscript writing and revision.

## Conflict of Interest Statement

The authors declare that the research was conducted in the absence of any commercial or financial relationships that could be construed as a potential conflict of interest.
